# Increased risk of atrial fibrillation among patients with bullous pemphigoid: a nationwide cohort study in Taiwan^☆^

**DOI:** 10.1016/j.abd.2026.501373

**Published:** 2026-06-03

**Authors:** Tai-Li Chen, Wan-Ting Huang, Chen-Yi Wu, Ching-Hui Loh, Huei-Kai Huang, Chi Ching-Chi

**Affiliations:** a Department of Dermatology, Taipei Veterans General Hospital, Taipei, Taiwan; b Department of Dermatology, School of Medicine, National Yang Ming Chao Tung University, Taipei, Taiwan; c Center for Clinical Epidemiology and Biostatistics, Hualien Tzu Chi Hospital, Buddhist Tzu Chi Medical Foundation, Hualien, Taiwan; d Department of Public Health, College of Medicine, National Yang Ming Chao Tung University, Taipei, Taiwan; e Center for Aging and Health, Hualien Tzu Chi Hospital, Buddhist Tzu Chi Medical Foundation, Hualien, Taiwan; f School of Medicine, College of Medicine, Tzu Chi University, Hualien, Taiwan; g Department of Family Medicine, Hualien Tzu Chi Hospital, Buddhist Tzu Chi Medical Foundation, Hualien, Taiwan; h Department of Dermatology, Chang Gung Memorial Hospital, Linkou Main Branch, Taoyuan, Taiwan; i School of Medicine, College of Medicine, Chang Gung University, Taoyuan, Taiwan

**Keywords:** Atrial fibrillation, Cohort study, Bullous pemphigoid

## Abstract

**Background:**

Bullous Pemphigoid (BP) and Atrial Fibrillation (AF) share common underlying mechanisms involving chronic inflammation, but their relationship was unclear.

**Objective:**

To assess the risk of incident AF in individuals with BP.

**Methods:**

This nationwide cohort study utilized Taiwan’s National Health Insurance Research Database from 2011 to 2019. Adults newly diagnosed with BP were identified and initially matched by age and sex to controls without BP. Stabilized inverse probability weighting was applied to balance baseline characteristics between cohorts. Cox proportional hazards models were used to estimate Hazard Ratios (HRs) and 95% Confidence Intervals (95% CIs) for incident AF. Subgroup analyses were performed by age and sex, along with several sensitivity analyses.

**Results:**

During the follow-up period, AF occurred in 510 of the 9,554 patients in the BP group and 1,902 of the 52,015 individuals in the non-BP group, corresponding to an incidence of 15.03 and 7.99 per 1,000 person-years, respectively. BP was associated with an increased risk of incident AF (HR 1.83; 95% CI 1.66–2.02). This association persisted across analyses stratified by age and sex. The findings were consistent across multiple sensitivity analyses.

**Study limitations:**

The unavailability of data on potential confounders such as lifestyle factors (smoking, alcohol, obesity) and disease severity.

**Conclusions:**

BP is linked to a heightened risk of AF. Prompt recognition of AF and timely cardiology referrals may be warranted in BP patients presenting with suggestive symptoms.

## Introduction

Bullous pemphigoid (BP) is a chronic autoimmune blistering dermatosis that predominantly affects the elderly, characterized by subepidermal blister formation and systemic inflammation.^1^, ^2^ Although primarily a dermatologic condition, emerging evidence suggests an association between BP and increased cardiovascular comorbidities, including heart failure and arrhythmias.^3^, ^4^ These associations may be mediated by chronic inflammation, immune dysregulation, and shared risk factors such as advanced age and comorbid conditions.^5^, ^6^

Atrial fibrillation (AF), the most common sustained cardiac arrhythmia, is strongly associated with systemic inflammatory states.^7^, ^8^ Inflammatory processes contribute to both structural and electrical remodeling of the atria, thereby increasing susceptibility to arrhythmogenesis.^9^, ^10^ Given the chronic systemic inflammation inherent in BP, a plausible pathophysiological link to AF exists. However, the association between BP and incident AF remains insufficiently investigated.

This nationwide cohort study aimed to evaluate the risk of incident AF in patients with BP by using a large-scale database and applying robust epidemiological methods to clarify this potential relationship.

## Methods

### Data source

This nationwide cohort study was conducted using claims data obtained from the National Health Insurance Research Database (NHIRD) of Taiwan. The NHIRD comprises comprehensive healthcare information on approximately 23.6 million individuals, representing over 99% of the Taiwanese population. Its validity and utility in epidemiological research have been well established in numerous published studies.^11^, ^12^, ^13^, ^14^, ^15^, ^16^, ^17^ A concise description of the NHIRD is provided in Note S1 in the Supplementary materials, with additional methodological details available in previously published literature.^18^, ^19^ Diagnostic and procedural information was identified using the International Classification of Diseases, Clinical Modification coding systems ‒ ICD-9-CM for data prior to 2016 and ICD-10-CM thereafter. The study protocol was reviewed and approved by the Research Ethics Committee of Hualien Tzu Chi Hospital (Approval nº IRB110-170-C). Given the anonymized and encrypted nature of the NHIRD data, the requirement for informed consent was formally waived. This study was conducted in accordance with the Strengthening the Reporting of Observational Studies in Epidemiology (STROBE) guidelines.

### Study population

All individuals aged ≥ 20-years who received a new diagnosis of BP from a dermatologist between 2011 and 2019 were considered eligible for inclusion. A diagnosis of BP was defined as either (1) at least three outpatient diagnoses within a one-year period or (2) A single inpatient discharge diagnosis, identified using ICD-9-CM code 694.5 or ICD-10-CM code L12. These diagnostic criteria have been previously validated and are associated with a high positive predictive value of 98%.^20^, ^21^ The authors excluded participants with a BP diagnosis before 2010 to ensure that newly diagnosed patients were recruited.

The exposed cohort consisted of patients with BP, while the unexposed cohort comprised individuals without BP. To prevent diagnostic overlap, the authors excluded subjects from the unexposed cohort who had documented diagnoses of other bullous dermatoses (ICD-9-CM codes: 694.0–694.4, 694.6, 694.8, 694.9; ICD-10-CM codes: L10, L11, L13, L14). Each patient with BP was matched by age and sex to four individuals from the unexposed cohort to initially construct exposure groups with comparable age and sex distributions. The index date for BP patients was established as the date of their first diagnosis. Controls in the unexposed cohort were assigned the same index date as their matched BP case. The authors excluded all individuals with a recorded history of AF preceding the index date.

### Outcomes

The primary outcome measured was new-onset AF (ICD-9-CM: 427.31; ICD-10-CM: I48.0, I48.1, I48.2, I48.91). The validity of these diagnostic codes for AF has been previously confirmed with a high positive predictive value.^22^ An outcome event was defined as a diagnosis made either at the outpatient or the inpatient department. All enrollees were followed from the index date until the occurrence of AF, death, or 31 December 2020 (the last date of the database), whichever came first.

### Covariates and confounders

From NHIRD reimbursement claims, the authors retrieved baseline demographic data. The authors identified and selected all comorbidities and medication use at baseline (see Tables S1 and S2 in the Supplementary materials) as potential confounders, consistent with prior studies.^23^, ^24^, ^25^ Pre-existing comorbidities were established through discharge diagnoses or diagnoses confirmed at least twice in outpatient services within one year preceding the index date, utilizing ICD-9-CM, ICD-10-CM, and procedure codes. The Charlson Comorbidity Index was applied to these pre-existing comorbidities to assess participants' overall systemic health status. Medication use at baseline was defined as a drug prescribed for a minimum of 30-days within the year prior to the index date. Monthly income levels were categorized based on NHI premiums, which are linked to income.

### Stratified analyses

Age- and sex-stratified analyses were conducted. For the age-stratified analysis, study subjects were divided into strata of < 65-years and ≥ 65-years, based on the overall mean age of the study population. For the sex-stratified analysis, participants were categorized into male and female strata according to a binary sex categorization in the database records.

### Statistical analyses

The authors utilized stabilized Inverse Probability Weighting (IPW), a propensity score-based method, to balance baseline characteristics between cohorts and mitigate potential confounding.^26^, ^27^ A propensity score was calculated for each patient using multivariable logistic regression models that incorporated all covariates listed in Table 1, thereby estimating the probability of BP exposure. Subsequently, stabilized IPW was performed individually for each comparison, encompassing overall, stratified, and sensitivity analyses, before the commencement of any data analysis. The application of stabilized weights is crucial for preventing the artificial inflation of sample size and for ensuring accurate variance estimation.^28^, ^29^ Although controls were initially selected using a 1:4 age- and sex-matching protocol to construct study groups with comparable age and sex distributions, subsequent analyses were conducted within a weighted cohort framework. This IPW approach generates a pseudo-population in which baseline covariates are balanced across study groups, and effect estimates therefore reflect weighted cohort comparisons.

**Table 1:** Demographic data of the study population after stabilized inverse probability weighting.

Characteristics^a^	BP cohort (n = 9,554)^a^	Non-BP cohort (n = 52,015)^a^	SMD^b^
**Mean age (SD), y**	76.6 (12.2)	78.0 (12.4)	0.109
**Sex (%)**			
Female	4734 (49.6)	24149 (46.4)	0.062
Male	4820 (50.5)	27866 (53.6)	0.062
**Income level (NTD) (%)**			
Financially dependent	4525 (47.4)	25826 (49.7)	0.046
15840‒24999	2649 (27.7)	15217 (29.3)	0.034
25000‒44999	1436 (15)	6683 (12.9)	0.063
≥ 45000	944 (9.9)	4289 (8.3)	0.057
**Mean Charlson Comorbidity Index (SD)**	1.5 (1.6)	1.7 (2.6)	0.085
**Comorbidities (%)**			
Diabetes mellitus	2418 (25.3)	14082 (27.1)	0.040
Hypertension	4376 (45.8)	21783 (41.9)	0.079
Stroke	1522 (15.9)	10456 (20.1)	0.109
Heart failure	454 (4.8)	2304 (4.4)	0.015
Coronary artery disease	1349 (14.1)	5743 (11)	0.093
COPD	995 (10.4)	6119 (11.8)	0.043
Chronic kidney disease	731 (7.7)	5330 (10.3)	0.091
Cirrhosis	100 (1.1)	561 (1.1)	0.003
Hyperlipidemia	2082 (21.8)	9615 (18.5)	0.083
Gout	493 (5.2)	2083 (4)	0.055
Malignancy	717 (7.5)	3359 (6.5)	0.041
Thyroid dysfunction	140 (1.5)	791 (1.5)	0.005
Dementia	1022 (10.7)	8306 (16)	0.155
Epilepsy	144 (1.5)	2171 (4.2)	0.161
Schizophrenia	45 (0.5)	654 (1.3)	0.085
Anxiety	678 (7.1)	2571 (4.9)	0.091
Depression	206 (2.2)	798 (1.5)	0.047
Bipolar disorder	98 (1)	385 (0.7)	0.031
Autoimmune disease	199 (2.1)	772 (1.5)	0.045
**Baseline medication use (%)**			
Antipsychotics	903 (9.5)	7198 (13.8)	0.137
Statins	2109 (22.1)	8856 (17)	0.128
ACEIs/ARBs	3271 (34.2)	16174 (31.1)	0.067
β-blockers	1873 (19.6)	7936 (15.3)	0.115
CCBs	2694 (28.2)	12363 (23.8)	0.101
Diuretics	1296 (13.6)	6465 (12.4)	0.034
NSAIDs	2076 (21.7)	7805 (15)	0.174
Corticosteroids	766 (8)	7609 (14.6)	0.210
PPIs	687 (7.2)	2961 (5.7)	0.061
Metformin	1390 (14.6)	7203 (13.9)	0.020
Sulfonylurea	1063 (11.1)	4573 (8.8)	0.078
TZDs	245 (2.6)	915 (1.8)	0.055
DPP-4 inhibitors	1148 (12)	6148 (11.8)	0.006
SGLT2 inhibitors	92 (1)	448 (0.9)	0.012
GLP-1 RAs	9 (0.1)	44 (0.1)	0.003
Meglitinide	258 (2.7)	1601 (3.1)	0.023
AGI	307 (3.2)	1449 (2.8)	0.025
Insulin	421 (4.4)	2754 (5.3)	0.042

AGI, Alpha-Glucosidase Inhibitors; ACEIs, Angiotensin-Converting Enzyme Inhibitors; ARBs, Angiotensin II Receptor Antagonists; BP, Bullous Pemphigoid; CCBs, Calcium Channel Blockers; COPD, Chronic Obstructive Pulmonary Disease; DPP-4, Dipeptidyl Peptidase-4; GLP-1 RAs, Glucagon-Like Peptide-1 Receptor Agonists; PPIs, Proton Pump Inhibitors; NSAIDs, Nonsteroidal Anti-Inflammatory Drugs; NTD, New Taiwan Dollar; SD, Standard Deviation; SGLT2, Sodium-Glucose Cotransporter-2; SMD, Standardized Mean Difference; TZDs, Thiazolidinediones.

A standardized mean difference of < 0.1 indicates a negligible difference.

a Weighted pseudo-population size generated by inverse probability weighting. The original sample size was presented in Table S3 in the Supplementary Materials.

b All covariates listed were used to calculate the propensity score for inverse probability weighting.

Baseline differences between the study cohorts were evaluated using the Standardized Mean Difference (SMD), with a value of < 0.1 signifying a negligible difference. Cox proportional hazards regression models, incorporating IPW, were employed to estimate Hazard Ratios (HRs). The authors estimated cumulative incidence curves using the Kaplan-Meier method with IPW. To ascertain differences in survival between BP and non-BP patients, a log-rank test was utilized. Statistical significance was set at a two-sided p-value < 0.01 due to the large sample size and multiple subgroup comparisons. All statistical analyses for this cohort study were conducted using SAS software (version 9.4; SAS Institute).

### Sensitivity analyses

To confirm the robustness of the primary analysis, the authors conducted multiple sensitivity analyses. First, if any covariate remained imbalanced between cohorts after stabilized IPW, the authors included it in the regression model for additional adjustment. Second, recognizing that mortality could be a competing risk, particularly for elderly patients, the authors utilized Fine-Gray subdistribution hazards models to estimate subdistribution HRs, with death considered as a competing risk event.^30^ Third, to determine whether the present research findings might be influenced or biased by potential extreme values in the weights, the authors performed an additional sensitivity analysis applying weight truncation: any weight exceeding 5 was reduced to this threshold.^31^

## Results

The authors initially included 61,569 subjects from the database. After applying stabilized IPW, the exposed cohort comprised 9,554 patients with BP, and the unexposed cohort included 52,015 controls without BP. Most baseline characteristics were appropriately balanced between the study cohorts after stabilized IPW (Table 1). The demographic data of study cohorts before stabilized IPW are provided in Table S3 in the Supplementary materials.

**Fig. 1:**
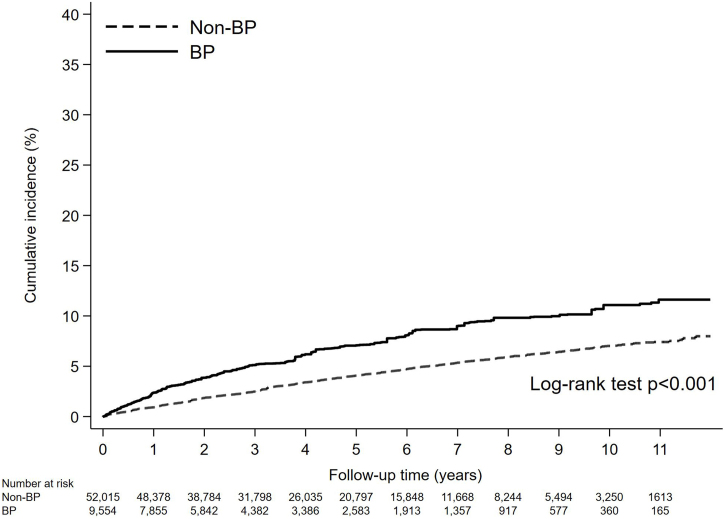
Cumulative incidence curve for incident atrial fibrillation. BP, Bullous Pemphigoid.

Overall, new-onset AF was identified in 510 of the 9,554 patients with BP, compared to 1,902 of the 52,015 controls, with incidence rates of 15.03 and 7.99 per 1,000 person-years, respectively (Table 2). The mean follow-up duration was 3.03-years. Patients with BP had a significantly increased risk of incident AF compared to controls (HR 1.83; 95% CI 1.66–2.02; p < 0.001), as shown in Table 2. Fig. 1 illustrates the cumulative incidence curves, with the BP cohort showing a higher cumulative incidence of developing AF (P < 0.001, log-rank test).

**Table 2:** Risk of incident atrial fibrillation among patients with bullous pemphigoid with sensitivity analyses.

Comparison (Main analysis)	Patients (n)	Events (n)	Person-years at risk	IR^a^	HR^b^	95% CI	P value
BP cohort	9554	510	33948	15.04	1.83	1.66–2.02	<0.001
Non-BP cohort	52015	1902	238010	7.99	1.00	Reference	

Sensitivity analyses				HR^b^	95% CI	P value
Additional adjustment for covariates with SMD > 0.1^c^				1.88	1.70–2.08	<0.001
Fine–Gray subdistribution hazard model				1.47	1.33–1.62	<0.001
IPW with weight truncation for weights exceeding 5				1.99	1.80–2.21	<0.001

BP, Bullous Pemphigoid; CI, Confidence Interval; HR, Hazard Ratio; IR, Incidence Rate.

a Incidence rate per 1000 person-years.

b The hazard ratios were calculated using a univariable Cox regression model with inverse probability weighting; the HR was calculated using the corresponding control cohort (patients without bullous pemphigoid) as the reference.

c Covariates with SMD > 0.1: age, stroke, dementia, epilepsy, antipsychotics, statins, β-blocker, calcium channel blockers, nonsteroidal anti-inflammatory drugs, and corticosteroids.

Table 3 presents the results of the age- and sex-stratified analyses. The increased risk of incident AF associated with BP remained significant in those aged ≥ 65-years (HR 1.98; 95% CI 1.79–2.19; P < 0.001) but not in those aged < 65-years (HR 1.61; 95% CI 1.01–2.58; P = 0.0467). When stratified by sex, the association between BP and increased AF risk also remained significant for both male (HR 2.46; 95% CI 2.14–2.83; P < 0.001) and female participants (HR 1.55; 95% CI 1.35–1.78; P < 0.001).

**Table 3:** Risk of incident atrial fibrillation among patients with bullous pemphigoid compared to controls, stratified by age and sex.

Outcomes	BP cohort		Non-BP cohort		HR^b^ (95% CI)	P value
	Events (n)	IR^a^	Events (n)	IR^a^		
**Age**						
<65 years	23	2.97	70	1.82	1.61 (1.01–2.58)	0.0467
≥65 years	493	18.6	1808	9.1	1.98 (1.79–2.19)	<0.001
**Sex**						
Female	254	15.9	1055	9.9	1.55 (1.35–1.78)	0.0055
Male	263	14.6	781	5.8	2.46 (2.14–2.83)	<0.001

BP, Bullous Pemphigoid; HR, Hazard Ratio; CI, Confidence Interval.

a Incidence rate per 1000 person-years.

b The hazard ratios were calculated using a univariable Cox regression model with inverse probability weighting; the HR was calculated using the corresponding control cohort (patients without bullous pemphigoid) as the reference.

As demonstrated in Table 2, sensitivity analyses consistently supported the primary findings. The sensitivity analysis, which included additional adjustment for covariates imbalanced after IPW, showed a similarly increased risk of AF among BP patients (HR = 1.88; 95% CI 1.70–2.08; P < 0.001). The analysis accounting for mortality as a competing risk, using Fine-Gray sub-distribution hazards models, also revealed a comparable result (HR = 1.47; 95% CI 1.33–1.62; P < 0.001). Furthermore, the sensitivity analysis that applied a truncation of weights exceeding 5 similarly yielded a consistent finding (HR = 1.99; 95% CI 1.80–2.21; P < 0.001).

## Discussion

The nationwide cohort study demonstrated that patients with BP had a 1.83-fold increased risk of developing AF compared with individuals without BP. A similar pattern of elevated AF risk was observed across age- and sex-stratified analyses, although the association in individuals aged < 65-years was not statistically significant under the applied threshold. Furthermore, multiple sensitivity analyses consistently supported the robustness of the association between BP and incident AF.

The elevated risk of AF observed among patients with BP may be attributed to the intricate interplay between chronic systemic inflammation and autoimmune-mediated pathophysiological mechanisms. BP is characterized by sustained immune activation and elevated circulating levels of proinflammatory cytokines such as interleukin-6, tumor necrosis factor-α, and interleukin-1β.^32^, ^33^ These cytokines promote atrial structural remodeling by stimulating fibroblast proliferation and extracellular matrix deposition, leading to atrial fibrosis and disruption of normal myocardial conduction pathways.^34^, ^35^ Concurrently, systemic inflammation induces oxidative stress and endothelial dysfunction, compromising the electrophysiological integrity of atrial myocytes and facilitating abnormal impulse generation and reentry.^36^ Moreover, autoimmune mechanisms, including molecular mimicry and potential cross-reactivity with cardiac antigens, may contribute to myocardial injury and electrical remodeling.^37^ Collectively, these processes establish a proarrhythmic substrate that predisposes individuals with BP to AF. These findings underscore the importance of proactive cardiovascular surveillance in this vulnerable patient population.

Previous studies have looked into the association of BP with comorbidities related to AF. A cohort study conducted by Yang et al. showed that BP have an increased risk of stroke (HR 2.37; 95% CI 1.78–3.15) and particularly ischemic stroke.^38^ One population-based cohort study from Denmark demonstrated that patients with autoimmune bullous disease have an elevated risk of arrhythmia (HR 1.16; 95% CI 1.02–1.32).^39^ In patients with pemphigus, one study by Namazi et al. indicated that the maximum P-wave duration and P-wave dispersion were significantly higher than those of the control group.^40^

To the best of our knowledge, this study is the first study to investigate the association between BP and AF. The main strength of the present research is the large-scale cohort study using nationwide data from real-world practice. Multiple sensitivity analyses were performed and confirmed our robust analyses with consistent results. However, our study has several limitations. First, as is inherent to registry-based studies, the NHIRD utilized for our analysis was limited by the lack of data on certain potential unmeasured confounders. Specifically, the authors were unable to adjust for lifestyle factors (e.g., smoking status, alcohol intake, obesity/body mass index) and granular clinical characteristics, such as the specific severity of BP or the amount of corticosteroid use. Furthermore, data on racial disparities were not captured, preventing an analysis of outcomes across different racial groups. Even though IPW was applied to minimize potential confounding effects, there is still a possibility of residual confounders. Second, the diagnoses of BP and HF relied primarily on ICD codes. While these codes have been shown to have high positive predictive value, potential misclassification might still have existed. Third, as the cohort was drawn from a single national database, the generalizability of these findings to other ethnic groups or geographic regions remains undetermined. Future studies involving diverse populations and varying healthcare settings are warranted to validate these results.

In conclusion, BP is associated with an increased risk for AF, as evidenced by this real-world cohort study. Cardiological evaluation should be considered for BP patients who present with symptoms such as palpitations, dyspnea, or lightheadedness. Electrocardiograms or echocardiography may be valuable tools for diagnosis and risk stratification in patients with BP. Recognizing BP as a potential condition necessitating heightened AF surveillance provides an opportunity for targeted interventions aimed at reducing the stroke burden.

## Authors' contributions

Tai-Li Chen: The study concept and design; data collection, or analysis and interpretation of data; statistical analysis; writing of the manuscript of critical review of important intellectual content; final approval of the final version of the manuscript.

Wan-Ting Huang: Data collection, or analysis and interpretation of data; statistical analysis; writing of the manuscript or critical review of important intellectual content; final approval of the final version of the manuscript.

Chen-Yi Wu: Data collection, or analysis and interpretation of data; writing of the manuscript or critical review of important intellectual content; final approval of the final version of the manuscript.

Ching-Hui Loh: Data collection, or analysis and interpretation of data; writing of the manuscript or critical review of important intellectual content; final approval of the final version of the manuscript.

Huei-Kai Huang: The study concept and design; data collection, or analysis and interpretation of data; statistical analysis; writing of the manuscript or critical review of important intellectual content; effective participation in the research guidance; final approval of the final version of the manuscript.

Ching-Chi Chi: The study concept and design; data collection, or analysis and interpretation of data; writing of the manuscript or critical review of important intellectual content; effective participation in the research guidance; final approval of the final version of the manuscript.

Tai-Li Chen, Huei-Kai Huang, and Ching-Chi Chi had full access to all the data in the study and take responsibility for the integrity of the data and the accuracy of the data analysis.

## Financial support

This work was supported by the Hualien Tzu Chi Hospital (TCRD112-024).

## Research data availability

The entire dataset supporting the results of this study was published in this article.

## Conflicts of interest

None declared.
