# Supplementary material for: Increased risk of atrial fibrillation among patients with bullous pemphigoid: a nationwide cohort study in Taiwan
Source: An Bras Dermatol. 2026 Jun 3;101(4):501373. doi: 10.1016/j.abd.2026.501373 (PMC13265673; doi:10.1016/j.abd.2026.501373)
Supplement: Supplementary file 1 [file mmc1.pdf]

ABD-D-25-00937

Supplementary Materials

Supplemental Note

Note S1. A brief introduction to Taiwan's National Health Insurance Research Database (NHIRD) P. 2

Supplemental Tables

Table S1. Diagnostic and procedure codes for covariates P. 3

Table S2. Codes for baseline medications P. 4

Table S3. Demographic data of the study population before stabilized inverse probability weighting P. 5

Supplemental Note 1 A brief introduction to Taiwan's National Health Insurance Research Database (NHIRD)

This retrospective cohort study utilized anonymized national health insurance data from Taiwan. Taiwan implemented compulsory universal health coverage (National Health Insurance [NHI] program) in 1995, managed by the government.[1] This system covers over 99% of the population, totaling approximately 23 million insured individuals. Taiwan's National Health Insurance Research Database (NHIRD) contains comprehensive data on demographics, healthcare claims, and medication information.[2] Until 2016, diagnoses and procedures were coded using International Classification of Diseases, Ninth Revision, Clinical Modification (ICD-9-CM). Since 2016, Taiwan has used the International Classification of Diseases, Tenth Revision, Clinical Modification (ICD-10-CM) codes. While the raw data is not publicly available to protect patient privacy (https://nhird.nhri.org.tw/en/Data_Protection.html), the study authors can provide access upon reasonable request in accordance with the data protection policy.

References

1. Hsing AW, Ioannidis JP. Nationwide Population Science: Lessons From the Taiwan National Health Insurance Research Database. JAMA Intern Med. 2015;175:1527–9.
2. Hsieh CY, Su CC, Shao SC, et al. Taiwan's National Health Insurance Research Database: past and future. Clin Epidemiol. 2019;11:349–58.

Table S1 Diagnostic and procedure codes for covariates.

	ICD-9-CM codes	ICD-10-CM codes
**Diabetes mellitus**	250	E08-E13
**Hypertension**	401-405	I10-I16
**Stroke**	430-438	I60-69
**Heart failure**	428	I50
**Coronary artery disease**	410-414	I20-25
**Chronic obstructive pulmonary disease**	490-492, 494, 496	J40-J44, J46
**Chronic kidney disease**	585	N18
**Cirrhosis**	571.2, 571.5, 571.6	K70.2, K70.3, K74
**Hyperlipidemia**	272	E78.0-78.5
**Gout**	274	M10
**Malignancy**	140-208, 209.0-209.3	C00-C96
**Pregnancy**	ICD-9-CM procedure 72–74 or ICD-9-CM 640.x1–676.x1, 640.x2–676.x2, and 650–659)	ICD-10-CM procedure 10E, 10P<br>ICD-10-CM O09-O92
**Thyroid dysfunction**	244.8,244.9,242, 245.2	E01.8, E02, E03.3, E03.8, E03.9, E05, E06.3
**Dementia**	290	F03
**Epilepsy**	345	G40
**Schizophrenia**	295	F20
**Anxiety**	300	F41
**Depression**	311, 296.2, 296.3, 296.5	F32
**Bipolar disorder**	296, 296.0, 296.1, 296.4, 296.6–296.9	F31
**Autoimmune disease**	340<br>555, 556.0–556.6, 556.8, 556.9<br>710.0<br>710.3, 710.4<br>710.2<br>710.1<br>714<br>446	G35<br>K50, K51<br>M32<br>M33<br>M35.0<br>M34<br>M06<br>M30.0, M30.1

ICD-9-CM, International Classification of Diseases, 9th Revision, Clinical Modification; ICD-10-CM, International Classification of Diseases, 10th Revision, Clinical Modification.

Table S2 Codes for baseline medications.

Medication	ATC codes
Antipsychotics	N05A
Statins	C10AA
Angiotensin-Converting Enzyme Inhibitors (ACEIs)/Angiotensin II Receptor Antagonists (ARBs)	C09
β-blocker	C07
Calcium Channel Blockers (CCBs)	C08
Diuretics	C03
Nonsteroidal Anti-Inflammatory Drugs (NSAIDs)	M01A
Corticosteroids	H02AB
Proton Pump Inhibitors (PPIs)	A02BC
Metformin	A10BA, A10BD02, A10BD03, A10BD04, A10BD05, A10BD07, A10BD08, A10BD10, A10BD11, A10BD13, A10BD14, A10BD15, A10BD16, A10BD17, A10BD18, A10BD20, A10BD22, A10BD23, A10BD25, A10BD26
Sulfonylurea	A10BB, A10BD02, A10BD06
Thiazolidinediones (TZDs)	A10BG, A10BD03, A10BD04, A10BD05, A10BD06, A10BD09, A10BD12, A10BD26
Dipeptidyl Peptidase 4 (DPP-4) inhibitors	A10BH, A10BD07, A10BD08, A10BD09, A10BD10, A10BD11, A10BD12, A10BD13, A10BD18, A10BD19, A10BD21, A10BD22, A10BD24, A10BD25
Sodium-Glucose cotransporter-2 (SGLT2) inhibitors	A10BK, A10BD15, A10BD16, A10BD19, A10BD20, A10BD21, A10BD23, A10BD24, A10BD25
Glucagon-Like Peptide-1 Receptor Agonists (GLP-1 RAs)	A10BJ
Meglitinide	A10BX02, A10BX03, A10BX08, A10BD14
Alpha-Glucosidase Inhibitors (AGI)	A10BF, A10BD17
Insulin	A10A

ATC codes, Anatomical Therapeutic Chemical codes.

Table S3 Demographic data of the study population before stabilized inverse probability weighting.

Characteristics[a]	BP cohort (n=11,450)	Non-BP cohort (n=45,800)	SMD[b]
**Mean age (SD), years**	77.3 (12.3)	77.3 (12.3)	0
**Sex (%)**			
Male	6,137 (53.6)	24,548 (53.6)	0
Female	5,313 (46.4)	21,252 (46.4)	0
**Income level (TWD), %**			
Financially dependent	3,368 (29.4)	27,524 (60.1)	0.6489
15,840–24,999	4,988 (43.6)	9,342 (20.4)	0.5126
25,000–44,999	1,821 (15.9)	5,451 (11.9)	0.1158
≥ 45,000	1,273 (11.1)	3,483 (7.6)	0.1211
**Mean Charlson Comorbidity Index (SD)**	2.65 (2.1)	0.68 (1.4)	1.047
**Comorbidities (%)**			
Diabetes mellitus	4,907 (42.9)	5,985 (13.1)	0.7036
Hypertension	7,269 (63.5)	12,602 (27.5)	0.7744
Stroke	4,546 (39.7)	2,569 (5.6)	0.8917
Heart failure	880 (7.7)	926 (2)	0.2661
Coronary artery disease	1,715 (15)	3,950 (8.6)	0.1981
Chronic obstructive pulmonary disease	2,249 (19.6)	2,163 (4.7)	0.4685
Chronic kidney disease	1,663 (14.5)	1,487 (3.3)	0.4041
Cirrhosis	148 (1.3)	232 (0.5)	0.0827
Hyperlipidemia	2,544 (22.2)	6,916 (15.1)	0.1835
Gout	598 (5.2)	1,347 (2.9)	0.1154
Malignancy	930 (8.1)	1,774 (3.9)	0.1797
Pregnancy	221 (1.9)	441 (1)	0.0813
Thyroid dysfunction	3,052 (26.7)	1,517 (3.3)	0.6923
Dementia	513 (4.5)	163 (0.4)	0.2705
Epilepsy	118 (1)	85 (0.2)	0.1080
Schizophrenia	804 (7)	1,855 (4.1)	0.1302
Anxiety	357 (3.1)	342 (0.8)	0.1727
Depression	165 (1.4)	222 (0.5)	0.0986
Bipolar disorder	231 (2)	428 (0.9)	0.0905
Autoimmune disease	4,907 (42.9)	5,985 (13.1)	0.7036
**Baseline medication use (%)**			
Antipsychotics	2,464 (21.5)	1,425 (3.1)	0.5836
Statins	2,869 (25.1)	6,583 (14.4)	0.2712
ACEIs/ARBs	4,915 (42.9)	9,475 (20.7)	0.4917
β-blockers	2,596 (22.7)	5,721 (12.5)	0.2699
Calcium channel blockers	3,973 (34.7)	7,859 (17.2)	0.4085
Diuretics	2,322 (20.3)	2,853 (6.2)	0.4235
Nonsteroidal anti-inflammatory drugs	1,907 (16.7)	6,017 (13.1)	0.0990
Corticosteroids	2,860 (25)	818 (1.8)	0.7244
Proton pump inhibitors	1,180 (10.3)	1,741 (3.8)	0.2563
Metformin	2,378 (20.8)	3,959 (8.6)	0.3476
Sulfonylurea	1,850 (16.2)	2,675 (5.8)	0.3344
Thiazolidinediones	395 (3.5)	634 (1.4)	0.1351
Dipeptidyl peptidase 4 inhibitors	2,868 (25.1)	2,356 (5.1)	0.5790
Sodium-glucose cotransporter-2 inhibitors	122 (1.1)	307 (0.7)	0.0431
Glucagon-like peptide-1 receptor agonists	19 (0.2)	28 (0.1)	0.0325
Meglitinide	653 (5.7)	526 (1.2)	0.2522
Alpha-glucosidase inhibitors	558 (4.9)	786 (1.7)	0.1772
Insulin	1,177 (10.3)	751 (1.6)	0.3712

ACEIs, angiotensin-converting enzyme inhibitors; ARBs, angiotensin II receptor antagonists; BP, bullous pemphigoid; TWD, New Taiwan Dollar; SD, standard deviation; SMD, standardized mean difference.

[a] All covariates listed were used to calculate the propensity score for inverse probability weighting.

[b] A standardized mean difference of < 0.1 indicates a negligible difference.
